# Reassessment of the Phylogeny and Systematics of Chinese *Parnassia* (Celastraceae): A Thorough Investigation Using Whole Plastomes and Nuclear Ribosomal DNA

**DOI:** 10.3389/fpls.2022.855944

**Published:** 2022-03-18

**Authors:** Ming-Ze Xia, Yan Li, Fa-Qi Zhang, Jing-Ya Yu, Gulzar Khan, Xiao-Feng Chi, Hao Xu, Shi-Long Chen

**Affiliations:** ^1^Key Laboratory of Adaptation and Evolution of Plateau Biota, Northwest Institute of Plateau Biology, Institute of Sanjiangyuan National Park, Chinese Academy of Sciences, Xining, China; ^2^University of Chinese Academy of Sciences, Beijing, China; ^3^School of Pharmacy, Weifang Medical University, Weifang, China; ^4^Qinghai Provincial Key Laboratory of Crop Molecular Breeding, Xining, China; ^5^Institute for Biology and Environmental Sciences, Carl von Ossietzky Universität Oldenburg, Oldenburg, Germany

**Keywords:** *Parnassia*, phylogeny, plastome, nuclear ribosomal DNA, divergence time, character evolution

## Abstract

*Parnassia* L., a perennial herbaceous genus in the family Celastraceae, consists of about 60 species and is mainly distributed in the Pan-Himalayan and surrounding mountainous regions. The taxonomic position and phylogenetic relationships of the genus are still controversial. Herein, we reassessed the taxonomic status of *Parnassia* and its intra- and inter-generic phylogeny within Celastraceae. To that end, we sequenced and assembled the whole plastid genomes and nuclear ribosomal DNA (nrDNA) of 48 species (74 individuals), including 25 species of *Parnassia* and 23 species from other genera of Celastraceae. We integrated high throughput sequence data with advanced statistical toolkits and performed the analyses. Our results supported the Angiosperm Phylogeny Group IV (APG IV) taxonomy which kept the genus to the family Celastraceae. Although there were topological conflicts between plastid and nrDNA phylogenetic trees, *Parnassia* was fully supported as a monophyletic group in all cases. We presented a first attempt to estimate the divergence of *Parnassia*, and molecular clock analysis indicated that the diversification occurred during the Eocene. The molecular phylogenetic results confirmed numerous taxonomic revisions, revealing that the morphological characters used in *Parnassia* taxonomy and systematics might have evolved multiple times. In addition, we speculated that hybridization/introgression might exist during genus evolution, which needs to be further studied. Similarly, more in-depth studies will clarify the diversification of characters and species evolution models of this genus.

## Introduction

*Parnassia* L. (Celastraceae) comprises approximately 60 perennial herbaceous species, and is widely distributed in the Arctic and temperate regions of the Northern Hemisphere (Phillips, 1982; Hultgard, 1987; Gu and Hultgård, 2001; Simmons, 2004). Most *Parnassia* species are endemic to China and have limited distribution, with >30 species only restricted to the Pan-Himalayan region (Hultgard, 1987), which is considered as an important diversity center of *Parnassia* (Handel-Mazzetti, 1941; Phillips, 1982; Ku, 1987; Wu, 2003; Simmons, 2004). The phylogenetic position of the genus is debatable, and prior studies showed that *Parnassia* should be treated as a member of Saxifragaceae (Hooker and Thomson, 1858; Engler, 1930; Cronquist and Takhtadzhian, 1981; Gu and Hultgård, 2001). However, *Parnassia* had also been suggested being closely related to Droseraceae morphologically (especially with the characteristics gynoecium; Pace, 1912; Dahlgren, 1980), to Hypericaceae (based on androecium and Chemotaxonomy through flavonoids; Arber, 1913; Jay, 1970), to Nymphaeaceae (based on vegetative characters; Hallier, 1901), and to Ochnaceae (based on peculiar staminodes; Bennett, 1869). Many systematists (e.g., Drude, 1875; Murbeck, 1918; Sharma, 1968; Klopfer, 1972; Hultgard, 1987) even suggested that *Parnassia* should be recognized as a separate family or order (e.g., Takhtadzhian and Takhtajan, 1997; Wu, 2003). The taxonomic debate over the genus seems to have ended over the last decades, especially with the advancement in molecular systematics. In contrast to the long-debated morphology-based systematics, more consistent results of the phylogenetic position of *Parnassia* were revealed by molecular studies, which suggested that *Parnassia* might be more closely related to Celastraceae than other families (Savolainen et al., 1997; Soltis et al., 1997, 2000; Soltis and Soltis, 1997; Simmons et al., 2001). Other studies subsequently confirmed this result, and *Parnassia* has been recovered as an early derived lineage of Celastraceae (Zhang and Simmons, 2006; Simmons et al., 2012; Bacon et al., 2016; Li et al., 2019). However, all these studies were based on a limited number of species, especially for those from its center of distribution and differentiation. Similarly, the molecular DNA markers used in these studies also lacked sufficient phylogenetic signals (Fischer and Steel, 2009; Philippe et al., 2011). Therefore, it is essential to provide molecular markers with sufficient informative characters to reconstruct the phylogenetic relationship of *Parnassia*.

Besides the debatable phylogenetic position of *Parnassia*, infrageneric systematics of the genus were also problematic. The distribution of *Parnassia* species in the Pan-Himalayan region often overlaps, and considerable morphological variation may occur within the same species. Scholars often have a different understanding of taxonomic traits, which led to different opinions about the infrageneric classification of the genus, including different ranks as section and series (Drude, 1875; Franchet, 1897; Engler, 1930; Handel-Mazzetti, 1941; Ku, 1987; Wu, 2003, 2005; Shu, 2017), and a plethora of taxonomic revisions (Turner and Veldkamp, 2001; Wu et al., 2004, 2008, 2009; Shu and Zhang, 2016, 2017; Wang et al., 2018; Yu et al., 2018; Ma et al., 2020; Dai et al., 2021). However, most of these investigations were based on morphological characters and need to be validated with molecular phylogenetic studies. Molecular phylogenetics for the genus based on nuclear ITS as well plastidial *trn*L-F and *trn*T-L markers suggested that *Parnassia* is monophyletic and that parallel evolution of morphological traits may occur (Wu, 2005). Yu (2019) reconstructed the phylogeny of *Parnassia* with 19 high polymorphic fragments of the plastid genome and suggested that the complete plastid genome dataset would be more comprehensive. Different studies have revealed that phylogenetic resolution and reliability seem to be related to the taxon as well as gene sampling (Wortley et al., 2005; Philippe et al., 2011). Since prior molecular phylogeny analyses on *Parnassia* are inadequate, there is an urgent need to construct a stable and precise phylogenetic tree to substantiate or negate the hypotheses made about *Parnassia* infrageneric systematics.

With the rapid development of next-generation sequencing technology, the amount of data used in phylogenetic research increased exponentially. Large-scale genomic data, such as plastid genome sequences and nuclear ribosomal DNA (nrDNA) data, can provide more phylogenetic informative sites than those of only a few DNA fragments. Recently, the plastid genome data has been successfully applied to resolve long-standing debates in phylogenetic positions of different taxa: e.g., the phylogenetic positions of Paeoniaceae within Saxifragales (Dong et al., 2018), Selaginellaceae (Zhang et al., 2019), and *sanguinolenta* group (Zhang H. R. et al., 2020). In addition, the plastid genome has also been employed to resolve the phylogenetic relationships of various plant lineages (Zhai et al., 2019; Liu et al., 2020; Ma et al., 2021). However, few plastid genomes have been sequenced for *Parnassia* and Celastraceae so far, which hinders the process of phylogenetic study of *Parnassia* (e.g., Wu, 2005; Yang et al., 2012).

With the development of molecular systematics, the phylogenetic research of *Parnassia* has made rapid progress. However, there is still a lack of large-scale data containing substantial phylogenetic signals (e.g., the plastid genome) for inferring the phylogenetic relationship of the genus. In addition, a large number of taxonomic revisions of *Parnassia* in recent years need to be confirmed by more reliable molecular systematics. Therefore, the present study attempts to resolve infrageneric phylogenetic questions about *Parnassia* using the whole plastid genomes and nrDNA (18S-ITS1-5.8S-ITS2-26S) data. The sampled species included seven of the nine sections of Chinese *Parnassia* (Ku, 1995; Wu, 2003) and covered the major species of *Parnassia* in the Pan-Himalayan region. The aims of this study are (1) to provide a robust and highly resolved phylogenetic tree for the phylogenetic position of *Parnassia*; and (2) to reassess the reliability of morphological features that have been widely used for taxonomic and phylogenetic considerations.

## Materials and Methods

### Sampling, Genome Sequencing, and Annotation

We sampled a total of 74 individuals, including 25 species (48 individuals) of *Parnassia* and 23 species (24 individuals) from all other 14 genera of Celastraceae, and two outgroups (Supplementary Table 1). To show the relationships within the species, we included two or more individuals from different localities for some species. Voucher specimens for the species sampled were deposited in the Qinghai-Tibetan Plateau Museum of Biology (HNWP), Northwest Institute of Plateau Biology, Chinese Academy of Sciences. Besides these 74 individuals, we retrieved 89 plastid genomes from the GenBank (Supplementary Table 2). In the final dataset, most orders of Superrosids were included, and two individuals of Asteraceae were selected as outgroups. We re-annotated the downloaded plastid genome sequence files using PGA (Qu et al., 2019) to ensure the accuracy of the gene coding sequences (CDSs) data obtained from GenBank in the analysis.

We extracted total genomic DNA using about 10 mg of silica-dried leaf tissue through modified CTAB protocols (Doyle and Doyle, 1987). The extracted DNAs of all the individuals were then sent to Novogene (Beijing, China). Genomic DNA library was generated using NEB Next^®^ Ultra™ DNA Library Prep Kit for Illumina (NEB, United States) following the manufacturer’s recommendations, and index codes were added to each sample and sequenced on an Illumina HiSeq 2500 sequencer (San Diego, CA, United States) using the paired-end option (2 × 150 bp). Sequencing quality was assessed through FastQC v. 0.11.8 (Andrews, 2010). Where necessary, we used Trimmomatic v. 0.33 (Bolger et al., 2014) to clean the sequencing data. The plastid genomes and nrDNA (18S-ITS1-5.8S-ITS2-26S) were assembled *de novo* using GetOrganelle (Jin et al., 2020) and then visually assembled, resulting in bandage v. 0.8.1 (Wick et al., 2015). Annotation of plastid genome was performed through the online program GeSeq^1^ (Tillich et al., 2017) with manual adjustment of start/stop codons and intron/exon borders in Sequin v. 15.50^2^. The nrDNA was annotated manually with the published ribosomal data of *Parnassia palustris* L. (AY929353), *Parnassia fimbriata* K. D. Koenig (AF036496), and *Trifolium repens* L. (MT735335).

### Sequence Alignment and Dataset Feature Evaluation

To reconstruct the phylogeny of *Parnassia* and its phylogenetic position among the Superrosids, we generated seven different datasets (datasets 1–7). We used 71 shared CDSs based on 88 annotated plastomes (83 plastomes from NCBI and five newly generated) to reassess the reliability of the morphological-based phylogenetic position of *Parnassia* (see details in Supplementary Table 3). Similarly, we used 73 shared CDSs data based on 80 annotated plastomes (six downloaded ones and the rest are newly generated) to reconstruct the infrageneric systematic of *Parnassia* (see details in Supplementary Table 3). To extract the shared CDSs, we used the program PhyloSuite v1.2.2 [see details in Zhang D. et al. (2020)]. We generated three different pairs of chloroplast datasets: the concatenated CDSs (datasets 1 and 4), utilizing only the first and second codon sites of the concatenated CDSs (datasets 2 and 5), and the third codon site of the concatenated CDSs (datasets 3 and 6). Additionally, we generated the nrDNA (18S-ITS1-5.8S-ITS2-26S; 5,879 bp) to explore the phylogenetic relationships of *Parnassia* (dataset 7). The alignment of datasets was conducted using MAFFT V.7.409 with the codon matching strategy (Katoh and Standley, 2013). To check the suitability of all the datasets, we performed the sequence substitution saturation test through DAMBE7 (Xia, 2018).

### Phylogenetic Analyses

We used the maximum likelihood (ML) and Bayesian inference (BI) statistics to reconstruct the *Parnassia* phylogenetic tree. Before tree reconstructions, we specified the GTR + G + I model inferred by jModelTest 2.1.6 (Darriba et al., 2012) under the Akaike information criterion (AIC) (Supplementary Table 4). For the ML analysis, we used the program RAxML 8.2.12 (Stamatakis, 2014), utilizing the option of 1,000 rapid bootstrap replicates. Similarly, BI analyses were carried out by MrBayes v 3.2.7a (Ronquist et al., 2012) implemented in the CIPRES Science Gateway V 3.3 (Miller et al., 2010). Each BI analysis was conducted with two independent runs and four Monte Carlo Markov chains (MCMCs) of ten million generations, and trees were sampled every 1,000 generations. The first 25% of the sampled trees were discarded as burn-in, and the remaining were used to generate the consensus tree and calculate the Bayesian posterior probabilities (PP).

### Molecular Dating Analyses and Ancestral Area Reconstruction

We used the concatenated CDSs of chloroplast (dataset 4) and estimated the divergence time of *Parnassia* utilizing the Bayesian statistics as implemented in BEAST v1.10.4 (Drummond et al., 2012). The parameters were set as GTR + I + Γ model and uncorrelated relaxed lognormal clock. The birth–death speciation model of the tree prior was selected according to the value of marginal likelihood, which was estimated by path sampling/stepping-stone sampling. The chain length of MCMC generations and the sampling frequency were set to 200,000,000 and 20,000, respectively. Generally, there may be many factors for the error in molecular clock analysis (e.g., taxon and gene sampling numbers, fossil calibrations strategy), which lead to bias in divergence time estimates (Sauquet et al., 2012; Bacon et al., 2016; Foster et al., 2017; Barba-Montoya et al., 2018). Due to the lack of reliable fossil evidence for the small perennial herbaceous plants of *Parnassia* (Coope et al., 1961), we selected four fossil records of its close relative taxa within Celastraceae as calibrations point to get a more reliable estimated divergence time (Figure 3). The strategies of divergence time estimation were as follows: Firstly, we selected the four most reliable evaluated fossils (Bacon et al., 2016) of relative taxa as fossil calibrations to avoid the generation of misleading results. Secondly, we employed a more informative dataset to reduce bias caused by a single gene (Zhai et al., 2019). We used four different times constraints based on the fossil records: (1) exponential priors (offset = 50.5, mean = 0.5) were set for the stem node of the monotypic *Catha* (following Poole and Wilkinson, 1999); (2) normal prior (mean = 26, St. dev. = 1) was set for the crown age of *Celastrus* + *Tripterygium* clade (following Xi et al., 2012; Bacon et al., 2016; Zhu et al., 2020); (3) exponential priors (offset = 56.5, mean = 0.833) were set for the stem node of *Glyptopetalum* + *Euonymus* (following Pitman and Rowan, 2012); and (4) secondary age was estimated from Xi et al. (2012) and Bacon et al. (2016), and a normal prior (mean = 90.6, St. dev. = 8.1) was set for the crown clade of Celastrales. We used BEAUti to generate the XML input files for BEAST, and the runs were performed on the CIPRES website (Miller et al., 2010). A total of four independent runs were followed in parallel, and the log files were combined using LogCombiner v1.10.4 (Drummond et al., 2012). We used Tracer v1.7 (Rambaut et al., 2018) to check the convergence of effective sample size (ESS). Lastly, the maximum clade credibility (MCC) tree with median heights was generated using TreeAnnotator v1.10.4 with the initial 10% trees discarded as burn-in. The final tree was visualized and edited using FigTree v.1.4.3^3^.

To assess the possible ancestral distribution regions of *Parnassia*, we performed an ancestral region reconstruction analysis in RASP v4.2 (Yu et al., 2020). The distribution regions of *Parnassia* were defined as belonging to the Pan-Himalayan and/or non-Pan-Himalayan. According to the distribution of species, we set the parameter “Max area” to two. The biogeographic models DEC, DIVALIKE, and BAYAREALIKE with/without J-parameter were tested by the R package “BioGeoBEARS” (Matzke, 2014), and the best-fit model was selected based on the Akaike Information Criterion (AIC) values.

## Results

### Plastomes and Nuclear Ribosomal DNA Assemblies

We obtained a total of 778.1 Gb sequencing data for all the 74 individuals with an average of 10.51 Gb ranging from 4.7 Gb (*Ribes glaciale* Wallich) to 15.86 Gb (*Parnassia nubicola* Wallich ex Royle) (Supplementary Table 1). The sizes of complete plastome ranged from 147,646 bp (*Saxifraga sinomontana* J. T. Pan & Gornall) to 163,053 bp (*Salacia obovatilimba* S. Y. Pao), and the sizes of nrDNA (18S-ITS1-5.8S-ITS2-26S) ranged from 5,783 bp (*Microtropis henryi* Merrill & F. L. Freeman) to 5,879 bp (*Parnassia foliosa* J. D. Hooker & Thomson). All the plastid genomes obtained in this study were composed of a typical quadripartite structure, similar to the previously published genomes of Celastraceae (Supplementary Figure 1). The total number of annotated genes varied between 113 and 115, including 79–81 protein-coding genes, four rRNA genes, and 30 tRNA genes, respectively. Overall, the GC content in these species ranged from 38.10% in *S. sinomontana* to 36.90% in *P. arborea* (Supplementary Table 1).

### Phylogenetic Position of *Parnassia*

**FIGURE 1:**
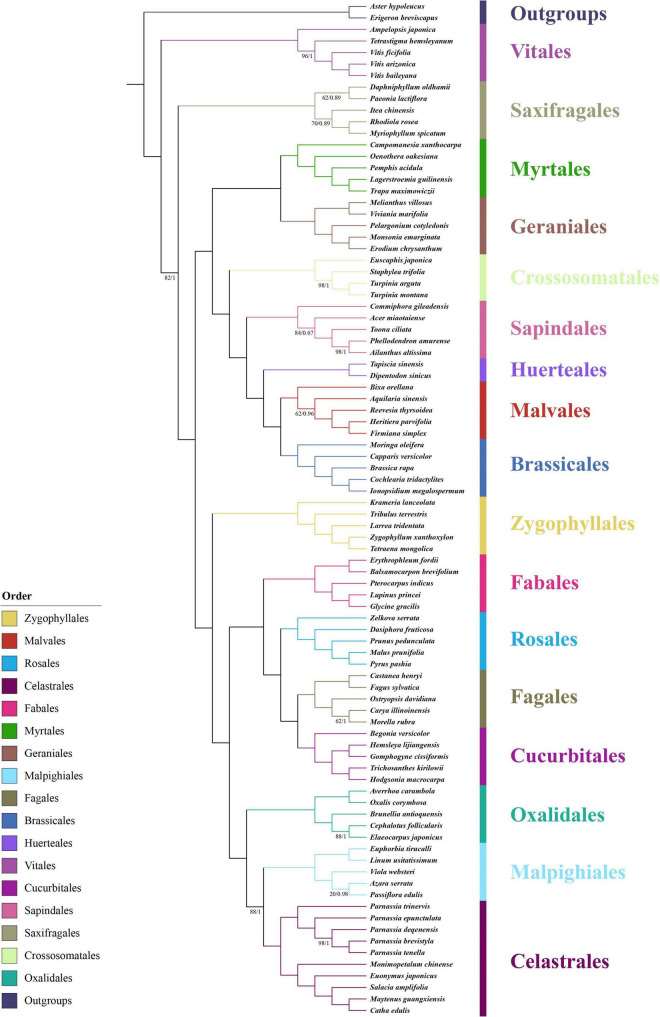
Phylogenetic position of the genus *Parnassia* in Superrosids revealed by the concatenated data sets of 71 CDSs. Numbers associated with branches are ML bootstrap values and Bayesian posterior probabilities. Nodes without numbers indicate 100% bootstrap support and 1.0 posterior probability.

The results about the suitability of the datasets used to investigate the phylogenetic position of *Parnassia* showed that the *I*_*ss*_ values were significantly smaller than the *I*_*ss–c*_ values (Supplementary Table 4). This suggested that these datasets had not reached substitution saturation and could be used for phylogenetic analyses (Xia et al., 2003). Though the phylogenetic trees were reconstructed with different datasets and different methods, all the results showed highly congruent topologies. Similarly, the relationships among different taxa were highly supported, suggesting that large-scale genomic data indeed significantly improved the resolution of phylogenetic trees (details see in Figure 1 and Supplementary Figures 2–5).

Our results showed that the phylogenetic relationships among all the 17 orders of Superrosids were strongly supported, with each clade corresponding to a tribe that was defined in previous studies. Celastrales, Malpighiales, and Oxalidales formed the COM clade; Cucurbitales, Fagales, Rosales, and Fabales clustered in the Fabid clade (nitrogen-fixing clade); COM, Fabid, and Zygophyllales formed the Fabidae; while Brassicales, Malvales, Huerteales, Sapindales, Crossosomatales, Geraniales, and Myrtales grouped into the malvid clade. Similarly, the Fabidae and malvid clades were sisters to one other. All the phylogenetic trees supported the above results, except the relationship among Saxifragales, Vitales, Fabidae, and malvid. In datasets (1 and 2), the relationship was resolved as (Fabidae, malvid), Saxifragales), Vitales, while in dataset 3, the relationship was resolved as [(Fabidae, malvid), Vitales], Saxifragales (Figure 1 and Supplementary Figures 2–5). Consistent with previous studies, *Parnassia* was clustered with the remaining genera of Celastraceae in all trees with fully supported (bootstrap support (BS) = 100, posterior probability (PP) = 1) (Figure 1 and Supplementary Figures 2–5).

### Phylogenetic Relationship of *Parnassia*

**FIGURE 2:**
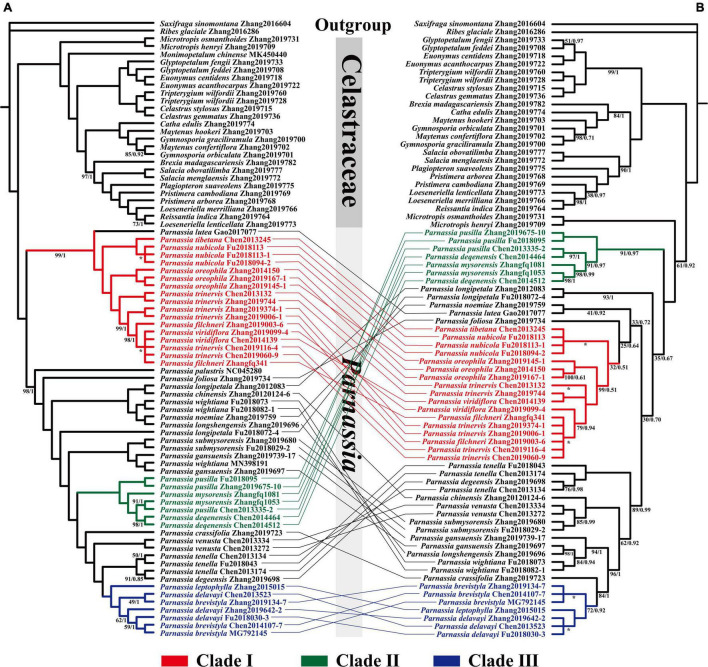
Phylogenetic relationship of *Parnassia* resolved based on 73 CDSs concatenated genes **(A)** and nrDNA marker **(B)**. Numbers associated with branches are ML bootstrap values and Bayesian posterior probabilities. Nodes without numbers indicate 100% bootstrap support and 1.0 posterior probability. Nodes with * indicate that the topological structure of ML tree and BI tree is inconsistent at this branch.

All the plastome datasets (datasets 4, 5, and 6) generated consistent trees with identical topologies of *Parnassia*, except for some uncertain clades (Figure 2 and Supplementary Figures 6–9). Phylogenetic trees showed that *Parnassia* was sister to the rest of Celastraceae with high support (BS = 100, PP = 1). In addition, all individuals of *Parnassia* were clustered together in all cases, indicating a well-supported monophyly of the genus. *Parnassia* sect. *Nectarotrilobos* Drude, the largest section in this genus, shows paraphyly, where some species of sect. *Nectarotrilobos* clustered with Clade I (*Parnassia lutea* Batalin, *Parnassia nubicola* Wallich ex Royle, *Parnassia oreophila* Hance, *Parnassia trinervis* Drude, *Parnassia viridiflora* Batalin, and *Parnassia filchneri* Ulbrich) and Clade II (*Parnassia pusilla* Wallich ex Arnott, *Parnassia mysorensis* F. Heyne ex Wight & Arnott, and *Parnassia deqenensis* T. C. Ku). However, the species of sect. *Nectarotrilobos* subsect. *Xiphosandra* (Franchet) Ku [*Parnassia brevistyla* (Brieger) Handel-Mazzetti, *Parnassia delavayi* Franchet, and *Parnassia leptophylla* Handel-Mazzetti] clustered together (Clade III). In addition, the species, *Parnassia chinensis* Franchet of sect. *Nectarotrilobos* was highly supported (BS = 100, PP = 1) as the sister to *Parnassia longipetala* Handel-Mazzetti of sect. *Saxifragastrum* Drude. Similarly, individuals of sect. *Nectaroquinquelobos* Ku (*Parnassia longshengensis* T. C. Ku and *Parnassia gansuensis* T. C. Ku) were not resolved as one clade, and so were the individuals of sect. *Saxifragastrum* Drude (*Parnassia tenella* Hooker & Thomson and *P. longipetala*) and sect. *Fimbripetalum* Drude (*Parnassia foliosa* Hooker & Thomson and *Parnassia noemiae* Franchet).

The topology of BI and ML analysis, reconstructed with the nrDNA sequence data, showed the same results. However, it was incongruent with the phylogeny retrieved by plastomes datasets (Supplementary Figures 6–9). In the nrDNA phylogenetic tree, the position of Clade I and Clade II was reversed (Figure 2). Three species of sect. *Nectarotrilobos* (*P. chinensis* (*Parnassia venusta* Jien, *Parnassia submysorensis* J. T. Pan) formed a highly supported clade (BS = 100, PP = 1). Similarly, *P. longshengensis*, *P. gansuensis*, and *Parnassia wightiana* Wallich ex Wight & Arnott were also recovered as a clade with enough support (BS = 94, PP = 1). Besides, concordant results of both the plastome-based and the nrDNA phylogenetic trees were shown in some branches (Figure 2), i.e., individuals of Clade I, II, and III were strongly recovered with the same topology in the nrDNA tree (except for *P. lutea* of Clade I).

### Divergence Time Estimates and Ancestral Range Inference

**FIGURE 3:**
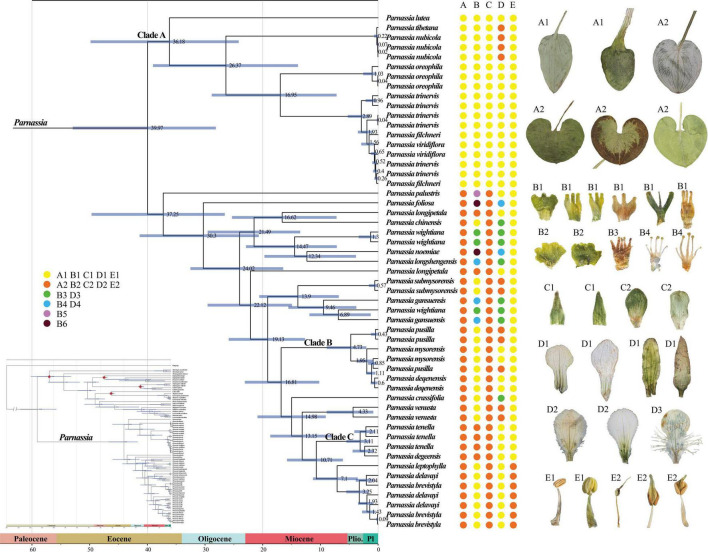
The dated phylogenetic tree of *Parnassia* is based on the dataset (4). Numbers associated with branches represent the mean estimated divergence time (Mya) and the blue bars correspond to the 95% highest posterior density (HPD) of divergence time. Red stars indicate four calibrating points. Pl, Pleistocene; Plio., Pliocene. **(A–E)** represent radical leaf, staminode, sepal, petal, and anther connective type, respectively. It should be noted that the characters shown in the figure only represent the characteristic types rather than the actual performance of the species. For a description of the specific feature types, please see Supplementary Table 5.

Bayesian relaxed molecular clock analyses suggested that the split between *Parnassia* and the remaining genera of Celastraceae occurred at 113.68 Mya, with the highest posterior density (HPD) intervals being 97.36–128.24 Mya (Figure 3). Within *Parnassia*, the first divergence occurred around 39.97 Mya (95% HPD = 28.13–52.93 Mya, Figure 3). The crown age of Clade A was inferred to be 36.18 Mya (95% HPD = 24.16–49.84 Mya, Figure 3). The mean divergence times of (*P. nubicola* and *P. tibetana*) and *P. oreophila* from the remaining species of Clade A were 26.37 Mya (95% HPD = 13.88–39.02 Mya) and 16.95 Mya (95% HPD = 7.20–28.83 Mya), respectively (Figure 3). The crown age of *P. palustris* was dated at 37.25 Mya (95% HPD = 26.57–49.67 Mya), followed by the crown age of *P. foliosa* at 30.30 Mya (95% HPD = 20.69–41.34 Mya) (Figure 3). The stem ages of Clade B were estimated to be 16.81 Mya (95% HPD = 10.19–23.09 Mya, Figure 3). The mean divergence times of Clade C and subsect. *Xiphosandra* were dated to 10.71 Mya (95% HPD = 6.10–15.78 Mya, Figure 3).

According to the AICc_wt value, the best model was BAYAREALIKE + J. The common ancestor of *Parnassia* probably originated in the Pan-Himalayan region (Supplementary Figure 10). The initial divergence of *Parnassia* took place at the Eocene and emerged as Clade A and Clade B. The diversification of Clade A was estimated to be in the non-Pan-Himalayan region, which was different from Clade B (Supplementary Figure 10).

## Discussion

Recently, high throughput sequence data has been used frequently to get robust topologies and to resolve the doubtful phylogenetic positions of different taxa [details see in Dong et al. (2018); Pouchon et al. (2018), Li et al. (2019); Zhai et al. (2019), Zhang H. R. et al. (2020)]. Here, we used the plastome and nrDNA datasets to deeply reassess the phylogenetic position of *Parnassia* within Superrosids and provide a resolved phylogenetic tree of its species. Our results highly supported the monophyly of *Parnassia*; however, the infrageneric systematics of the genus showed incongruencies.

### Phylogenetic Position of *Parnassia* in Superrosids

We reconstructed a well-resolved phylogenetic tree for 17 orders of Superrosids. The COM clade topology of all the phylogenetic trees revealed that Celastrales was sister to Malpighiales with high-support, and then this clade is sister to Oxalidales. The COM clade topology (Figure 1) obtained here was consistent with Zhang and Simmons (2006); Soltis et al. (2007), and Li et al. (2019), nevertheless, incongruent with many other previous studies (Qiu et al., 2010; Soltis et al., 2011; Ruhfel et al., 2014; Chen et al., 2016; Group et al., 2016; Sun et al., 2016). Although the sequence data used for phylogenetic analysis of COM clade has significantly increased in recent years, their systematics are still problematic or at least did not obtain well-supported topologies [details see in Li et al. (2019) and here in this study].

The phylogenetic trees based on different datasets to investigate the phylogenetic position of Saxifragales and Vitales were incongruent (Figure 1 and Supplementary Figures 2–5). The phylogenetic trees of dataset 3 recovered a similar topology as proposed by Li et al. (2019; Supplementary Figures 4, 5). However, the trees of datasets 1 and 2 showed reverse topologies of the phylogenetic position between Saxifragales and Vitales (Supplementary Figures 1–3). All the phylogenetic trees revealed that *Parnassia* was closely related to Celastrales (Celastraceae: Figure 1 and Supplementary Figures 2–5). Therefore, our results rejected the hypothesis based on morphology, which considered *Parnassia* was close to Saxifragaceae or other families (e.g., Hooker and Thomson, 1858; Hallier, 1901; Pace, 1912; Arber, 1913; Engler, 1930; Thorne, 1968; Spongberg, 1972; Bensel and Palser, 1975; Dahlgren, 1980; Cronquist and Takhtadzhian, 1981; Ku, 1987; Gu and Hultgård, 2001). Previous studies have questioned the relationship between *Parnassia* and Saxifragaceae. Grund and Jensen’s (1981) comparative investigations of serological characteristics of seed proteins revealed that there was no relationship between *Parnassia* and Saxifragaceae. In addition, Matthews and Endress (2005) compared the floral structures of Celastrales, expounded 15 shared floral features between *Parnassia* and Celastraceae, and described five different floral structural characteristics that distinguished *Parnassia* from Saxifragaceae. Soltis et al. (1990) found that the relationship between *Parnassia* and *Brexia* was closer than Saxifragaceae by using *rbc*L. It was also confirmed in other later molecular phylogenetic researches (Chase et al., 1993; Morgan and Soltis, 1993; Soltis et al., 1997, 2000; Soltis and Soltis, 1997; Koontz and Soltis, 1999). Similarly, our results corroborated that *Parnassia*, belonging to Celastrales, was closely related to Celastraceae.

To investigate the phylogenetic position of *Parnassia*, we sampled species of *Parnassia* and 15 genera of Celastrales distributed in China and reconstructed the phylogenetic relationship. Two families were included in Celastrales: Celastraceae and Lepidobotryaceae. In previous investigations, *Parnassia* and Celastraceae were resolved as one clade according to the *rbc*L (Savolainen et al., 1997), and Lepidobotryaceae was sister to this clade (also see Zhang and Simmons, 2006). The floral morphological study revealed that Lepidobotryaceae was more distant from *Parnassia* and Celastraceae, and these three taxa were also supported as a group with some shared floral features (Matthews and Endress, 2005). Nevertheless, the relationship between *Parnassia* and Celastraceae was still controversial (details see introduction). In this study, plastome phylogenetic suggested that *Parnassia* was sister to Celastraceae with high support, which was consistent with the results of Simmons et al. (2012), Bacon et al. (2016), Chen et al. (2016), and Li et al. (2019). However, the nrDNA data clustered *Parnassia* and *Microtropis* together and then resolved them as sister groups to the other genera of Celastraceae (also see Simmons et al., 2012 and Sun et al., 2016). Discordance between the plastid genome and nrDNA analyses is a common phenomenon in molecular phylogenetic studies (see Lee-Yaw et al., 2019; Chen et al., 2020; Liu et al., 2020; Ma et al., 2021; Uckele et al., 2021), which was usually suggested to be a result of horizontal gene transfer (Stegemann et al., 2012), hybridization (Rieseberg et al., 1991, 1996) and incomplete lineage sorting (Degnan and Rosenberg, 2009).

In addition to the doubtful phylogenetic relationship between *Parnassia* and *Microtropis* shown in this study, the relationship among *Parnassia*, *Perrottetia*, and *Mortonia* is also unclear. Previous studies revealed that (*Parnassia*, *Lepuropetalon*) + (*Perrottetia*, *Mortonia*) were sister to other genera of Celastraceae, suggesting that *Parnassia* should be considered as an early derived lineage of Celastraceae (Simmons et al., 2001; Sun et al., 2016). Meanwhile, several studies demonstrated that *Parnassia* was sister to all the remaining genera of Celastraceae (Simmons et al., 2012; Bacon et al., 2016; Chen et al., 2016; Li et al., 2019). Here, it was suggested that the topology of trees based on plastome have higher resolution in comparison with the nrDNA. With the combination of the previous studies and our results of the plastome tree, we speculated that *Parnassia* was a basalmost taxon of Celastraceae. Nevertheless, it is still not mature enough to make clearer hypotheses before resolving the phylogenetic relationships among *Parnassia*, *Mortonia*, *Perrottetia*, and *Microtropis*.

### Biogeography and Divergence Estimation of Chinese *Parnassia*

The divergence time in this study is congruent with the previous geographical study of *Parnassia* (Wu, 2005). However, due to the limited fossil calibrations points (lack of reliable fossil records of *Parnassia*), the results of divergence estimation in this study should be treated cautiously. Wu (2005) discussed the geographical distribution of *Parnassia* and supposed that its origin was in the early tertiary periods or even earlier. Bell et al. (2010) estimated the divergence times of key angiosperm lineages and showed that the crown-group age of *Parnassia* was estimated as 29 (17–43) Ma (exponential priors) and 34 (19–48) Ma (Lognormal priors). Here, the diversification of *Parnassia* was estimated to be 39.97 Mya (95% HPD = 28.13–52.93 Mya, Figure 3), which was earlier than the result of Bell et al. (2010; 34 Mya, 95% HPD = 19–48 Mya). The limited taxon coverage may reduce the accuracy of divergence time estimation [only one individual representing *Parnassia* in Bell et al. (2010) and Gao et al. (2017)], which may partially account for the slight inconsistency.

Most species in this study were distributed in the southeastern Qinghai-Tibetan Plateau and its surrounding mountainous regions. The ancestral area inference results indicated that the ancestral lineage of *Parnassia* probably originated in the Pan-Himalayan region. The north of Tibet localized uplift to the near present heights during the Eocene, while northwestern Yunnan formed its present topography during the late Eocene/early Oligocene (Spicer et al., 2021). Our results of divergence time estimation indicated that the genus *Parnassia* began to diverge and spread to non-Pan-Himalayan areas during the Eocene. Combining with the Qinghai-Tibetan Plateau uplift, the global climate oscillations led to a significant change in plant diversity during the Eocene–Oligocene transition period (Abels et al., 2011; Deng et al., 2020). As a result, the differentiation *Parnassia* species began to appear at Oligocene in the Pan-Himalayan region, and then mountain building and Asian monsoon may have combined to promote species diversification and colonization in the early to middle Miocene (Xing and Ree, 2017; Ding et al., 2020). Finally, the final uplift and climate oscillation of the Qinghai-Tibet Plateau in the late Miocene or Pliocene has jointly driven the rapid diversification of *Parnassia* species (Chen et al., 2012; Zhou et al., 2013). This may partially explain the high species richness of *Parnassia* species in the Pan-Himalayan region. Similar to previous studies in this space–time (Sun et al., 2017; Lu et al., 2018), it is probable that the large-scale expansion occurred after species diversification of *Parnassia* and accelerated the colonization of adjacent areas.

### Infrageneric Relationships of *Parnassia*

We mainly focused on resolving the phylogenetic relationship of *Parnassia* species and advancing some supportive hypotheses about its systematics. Our results recovered a well-supported monophyletic clade of *Parnassia* by using both the plastome and the nrDNA datasets (Figure 2). Notably, most of the plastome-based topology branches reached a 100% support rate, which significantly improved the phylogenetic resolution (Figure 2).

The molecular systematics of *Parnassia* have focused on the phylogenetic position problems, and there were few studies on the phylogenetic relationship within this genus (Wu, 2005; Yang et al., 2012; Yu, 2019). In this study, more comprehensive plastome and nrDNA data were used to elaborate the phylogeny of *Parnassia* for the first time. Consistent with previous studies, *Parnassia* has been fully supported as a monophyletic group. Clade I (*P. lutea*, *P. tibetana*, *P. nubicola*, *P. oreophila*, *P. trinervis*, *P. filchneri*, and *P. viridiflora*) consisted of seven species of sect. *Nectarotrilobos*, forming a monophyletic branch (details see in Wu, 2005). *Parnassia lutea*, without the cauline leaves, was considered to represent the early colonizer of sect. *Nectarotrilobos* to high-altitude areas (Wu, 2003, 2005). Although *P. lutea* was located at the base of Clade I, reflecting it was an earlier element of Clade I, our results did not support its more ancient hypothesis within the genus. Ma et al. (2020) proposed that *P. tibetana* was synonymous to *P. nubicola.* Likewise, *P. tibetana* has been resolved as nested within the individuals of *P. nubicola*, and we did not find any significant molecular differences between *P. tibetana* and *P. nubicola* (Figure 2). Compared to our results of *P. oreophila*, Yu (2019) also demonstrated that *P. oreophila*, *P. nubicola*, *P. laxmannii*, *P. trinervis*, and *P. cabulica* belonged to the same clade. As inferred from the distribution range and divergence time, *P. trinervis*, *P. filchneri*, and *P. viridiflora* may be the species differentiated from *P. oreophila* in the process of colonizing to high altitude during Miocene. Hence, we herein supported the taxonomic revisions of *P. tibetana* and *P. nubicola* in Ma et al. (2020). Individuals of *P. trinervis*, *P. filchneri*, and *P. viridiflora* were nested within each other (Figure 2). During field investigation, we found that the distribution range of *P. trinervis* overlapped with *P. viridiflora*. Furthermore, the petal color of these two species changed from dark green to light green and white, and even different colors might occur in the same population (petal color was the key character to distinguish these two species). Therefore, we supported the recommendation that *P. viridiflora* should be treated as the synonym of *P. trinervis* by Yang et al. (2012) [also in Shu et al. (2017)]. For *P. filchneri*, a follow-up study is recommended to assess the relationship with *P. trinervis*. Previous studies suggested that *P. palustris* appeared earlier than other species of *Parnassia* and should be a relict species (Wu, 2003, 2005). Divergence time estimation supported this speculation (Figure 3), and we suggested that *P. palustris* was the ancestor species of Pan-Himalayan region.

Clade II consisted of three species of sect. *Nectarotrilobos* (*P. pusilla*, *P. mysorensis*, and *P. deqenensis*). For *P. deqenensis*, Wu (2005) suggested reducing “it” as the synonym of *P. trinervis*, while Shu et al. (2017) proposed reducing “it” as the synonym of *P. pusilla.* Our results here showed that the three species in Clade II were grouped into one branch in all trees, while individuals were nested within each other in phylogenetic trees (Figure 2). Based on the above results, we agreed with the taxonomic revision of Shu et al. (2017). Meanwhile, further studies with *P. mysorensis* and *P. pusilla* are suggested.

Clade III consisted of three species of sect. *Nectarotrilobos* subsect. *Xiphosandra* (*P. brevistyla*, *P. delavayi*, and *P. leptophylla*). Yu et al. (2018) found that the continuous characteristics were mistakenly used in distinguishing these three species. Hence, *P. brevistyla* and *P. leptophylla* were designated as synonyms of *P. delavayi*. In this study, the results of phylogenetic relationships showed that individuals of these three species were nested within each other (Figure 2), supporting the taxonomic revision of Yu et al. (2018).

There were widespread inconsistencies in the topological structure of the plastid trees and the nrDNA trees (Figure 2), such as the systemic position of *P. noemiae*, *P. lutea*, *P. foliosa*, *P. chinensis*, *P. venusta*, *P. submysorensis*, *P. longshengensis*, *P. gansuensis*, and *P. wightiana* were changed. The individuals of *P. wightiana* were resolved as polyphyletic in plastid trees (Figure 2), which could be attributed to the previously reported hybridization of this species (Yu, 2019). Furthermore, *P. longipetala* was not monophyletic as well in plastid trees, but clustered together in nrDNA trees, suggesting that incomplete lineage sorting, hybridization, or gene introgression events might have occurred with its related sympatric species. After comparing with many previous studies about the genus (e.g., Chen et al., 2020; Liu et al., 2020; Ma et al., 2021; Uckele et al., 2021), we speculated that widespread hybridization or introgression events might occur in *Parnassia* species distributed in and around the Hengduan Mountains (e.g., *P. venusta*, *P. longshengensis*, *P. wightiana*, *P. submysorensis*, and *P. chinensis*).

Wu (2005) and Yu (2019) explored the phylogeny of *Parnassia* based on morphology. However, the results showed that almost all characters (such as the shape of petal margin and the number of staminodes lobs) that have been used for taxonomy and systematic might have undergone parallel or even reverse evolution [details in Shu (2017)]. Our phylogenetic results showed that except for the characters of basal leaves and anther connective, other characteristics may not represent the evolutionary relationship of *Parnassia* (Figure 3). The sect. *Nectarotrilobos*, sect. *Saxifragastrum*, sect. *Fimbripetalum*, and sect. *Nectaroquinquelobos* were resolved as polyphyletic. Shu (2017) proposed reducing *P. venusta* and *Parnassia degeensis* as synonyms of *Parnassia cacuminum.* However, in this study, individuals of *P. venusta* and *P. degeensis* were not clustered together, which was inconsistent with traditional taxonomy revisions (Shu, 2017). The molecular phylogenetic analyses showed that a single species with continuous quantitative traits might be divided into several different species in the morphological study of *Parnassia*, e.g., *P. brevistyla*, *P. delavayi*, and *P. leptophylla* (Yu et al., 2018). In addition, different species with similar characters may be merged into one species, e.g., *P. venusta*, *P. degeensis*, and *P. cacuminum* (see details in Figure 3; Shu and Zhang, 2017). Characters (e.g., staminodes 3-lobed) of *Parnassia* might evolve at multiple times, and introgression or hybridization events might have happened in the process of evolution. This may explain why the trend of morphological character evolution of *Parnassia* has always been controversial in the traditional taxonomy.

## Conclusion

Here we utilized the whole plastome and nrDNA (5,879 bp) markers to reconstruct the position of *Parnassia* within the Superrosids and phylogenetic relationships of Chinese *Parnassia* species. Our results corroborated that *Parnassia* is most closely related to Celastraceae, while follow-up studies are still needed to determine the relationship between *Parnassia* and several related genera. However, there was incongruence between the plastome and nuclear phylogenetic trees. In addition, we presented a first attempt to use the fossil records of Celastraceae to estimate the divergence of Chinese *Parnassia*, which was strongly consistent with the expected results of previous biogeographic studies. Moreover, the molecular phylogenetic results confirmed numerous taxonomic revisions and suggested that many traits have been widely used for classification, and that systematic considerations were the results of multiple evolutions. Our results will provide valuable insights into the taxonomic study of *Parnassia* and expand the foundation for further exploration of the evolutionary diversity of the genus.

## Data Availability Statement

The datasets presented in this study can be found in online repositories. The names of the repository/repositories and accession number(s) can be found in the article/Supplementary Material.

## Author Contributions

F-QZ: conceptualization, funding acquisition, and resources. S-LC: funding acquisition, resources, and supervision. M-ZX: formal analysis, data curation, and writing – original draft. YL: resources, formal analysis, and writing – review and editing. J-YY, X-FC, and HX: resources and formal analysis. GK: formal analysis and writing – review and editing. All authors contributed to the article and approved the submitted version.

## Conflict of Interest

The authors declare that the research was conducted in the absence of any commercial or financial relationships that could be construed as a potential conflict of interest.

## Publisher’s Note

All claims expressed in this article are solely those of the authors and do not necessarily represent those of their affiliated organizations, or those of the publisher, the editors and the reviewers. Any product that may be evaluated in this article, or claim that may be made by its manufacturer, is not guaranteed or endorsed by the publisher.

## Footnotes

1
http://chlorobox.mpimp-golm.mpg.de/geseq.html

2
https://www.ncbi.nlm.nih.gov/guide/data-software

3
http://tree.bio.ed.ac.uk/software/figtree/

## Supplementary Material

Supplementary Figure 1 Representation of the chloroplast genome map of *Parnassia trinervis*. The genome was plotted using Chloroplot (https://irscope.shinyapps.io/Chloroplot/).

Supplementary Figure 2 Phylogenetic position of *Parnassia* in Superrosids reconstructed with the dataset based on the first and second sites on the concatenated codons. Numbers associated with branches are ML bootstrap values. Nodes without numbers indicate 100% bootstrap support.

Supplementary Figure 3 Phylogenetic location of *Parnassia* in Superrosids reconstructed with the dataset based on the first and second sites on the concatenated codons. Numbers associated with branches are Bayesian posterior probabilities. Nodes without numbers indicate posterior probability of 1.

Supplementary Figure 4 Phylogenetic position of *Parnassia* in Superrosids recovered with the dataset consists of third site on the codons in concatenated. Numbers associated with branches are ML bootstrap values. Nodes without numbers indicate 100% bootstrap support.

Supplementary Figure 5 Phylogenetic position of *Parnassia* in Superrosids recovered with the dataset consists of third site on the codons in concatenated. Numbers associated with branches are Bayesian posterior probabilities. Nodes without numbers indicate 1.0 posterior probability.

Supplementary Figure 6 Phylogenetic relationship of *Parnassia* species resolved with the dataset of first and second sites on the concatenated codons. Numbers associated with branches are ML bootstrap values. Nodes without numbers indicate 100% bootstrap support.

Supplementary Figure 7 Phylogenetic relationship of *Parnassia* species resolved with the dataset of first and second sites on the concatenated codons. Numbers associated with branches are Bayesian posterior probabilities. Nodes without numbers indicate 1.0 posterior probability.

Supplementary Figure 8 Phylogenetic relationship of *Parnassia* species resolved with the dataset consists of third site on the codons in concatenated. Numbers associated with branches are ML bootstrap values. Nodes without numbers indicate 100% bootstrap support.

Supplementary Figure 9 Phylogenetic relationship of *Parnassia* species resolved with the dataset consists of third site on the codons in concatenated. Numbers associated with branches are Bayesian posterior probabilities. Nodes without numbers indicate 1.0 posterior probability.
