# Supplementary figures and images for: Reassessment of the Phylogeny and Systematics of Chinese Parnassia (Celastraceae): A Thorough Investigation Using Whole Plastomes and Nuclear Ribosomal DNA
Source: Front Plant Sci. 2022 Mar 18;13:855944. doi: 10.3389/fpls.2022.855944 (PMC8971841; doi:10.3389/fpls.2022.855944)

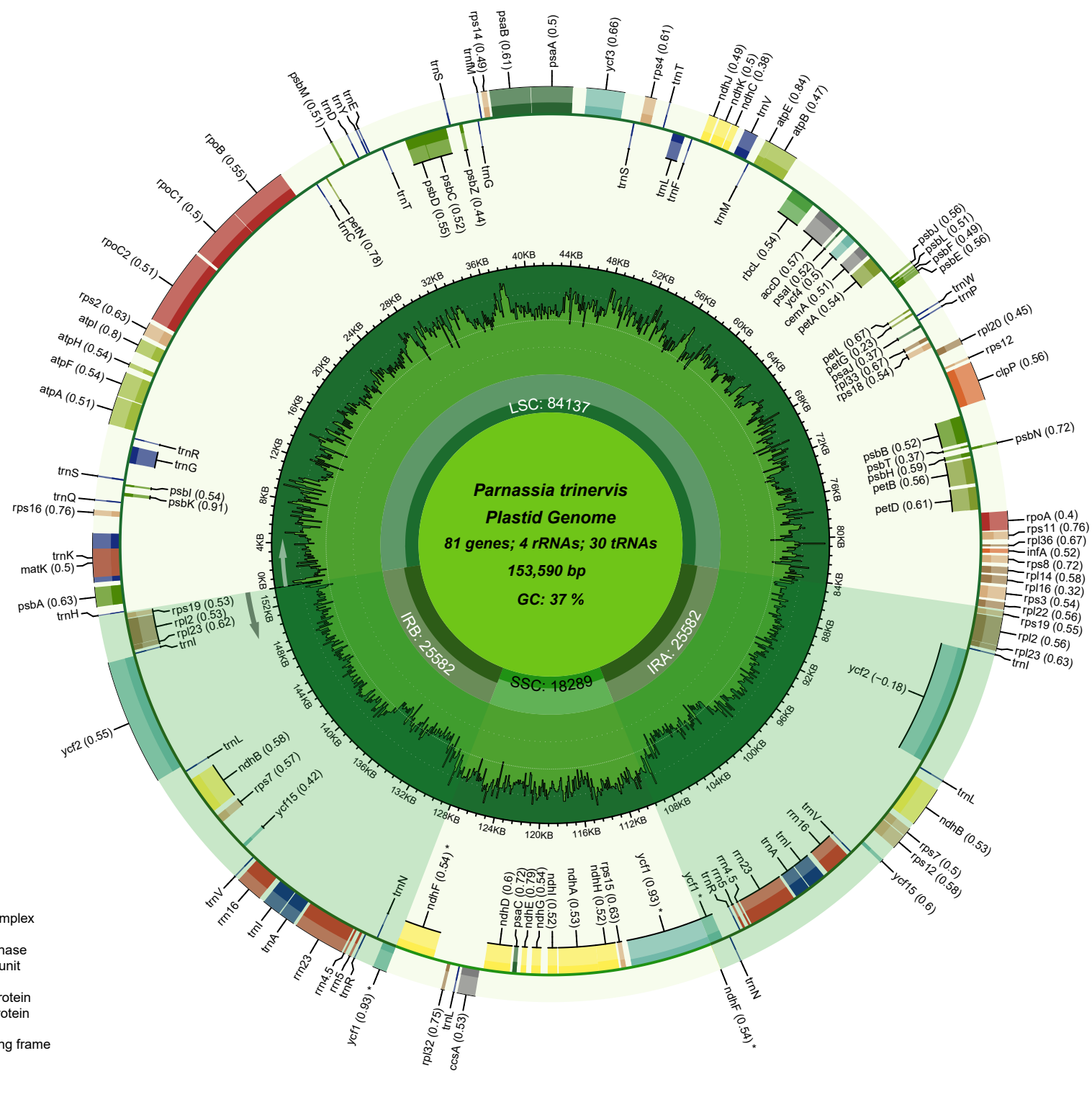

Supplement: Supplementary Figure 1 — Representation of the chloroplast genome map of Parnassia trinervis. The genome was plotted using Chloroplot (https://irscope.shinyapps.io/Chloroplot/). [file Image_1.PDF]

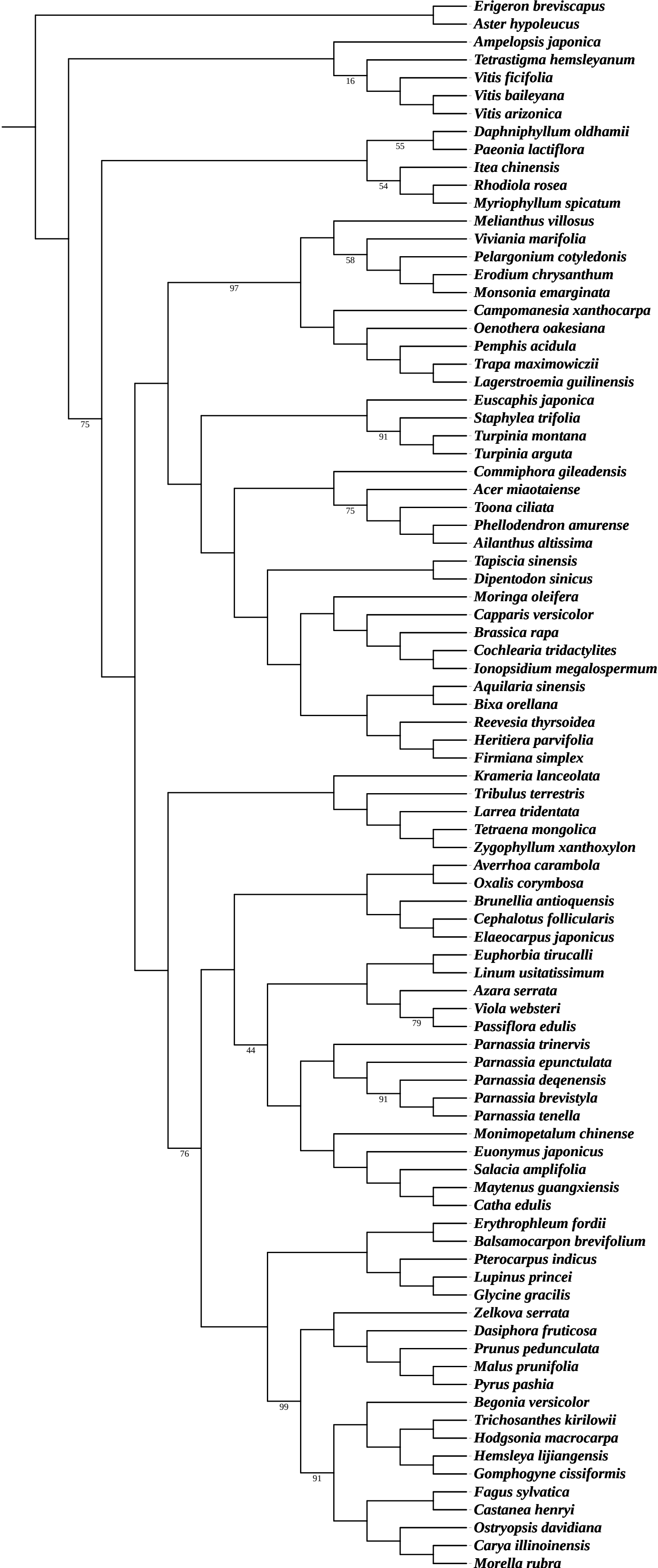

Supplement: Supplementary Figure 2 — Phylogenetic position of Parnassia in Superrosids reconstructed with the dataset based on the first and second sites on the concatenated codons. Numbers associated with branches are ML bootstrap values. Nodes without numbers indicate 100% bootstrap support. [file Image_2.PDF]

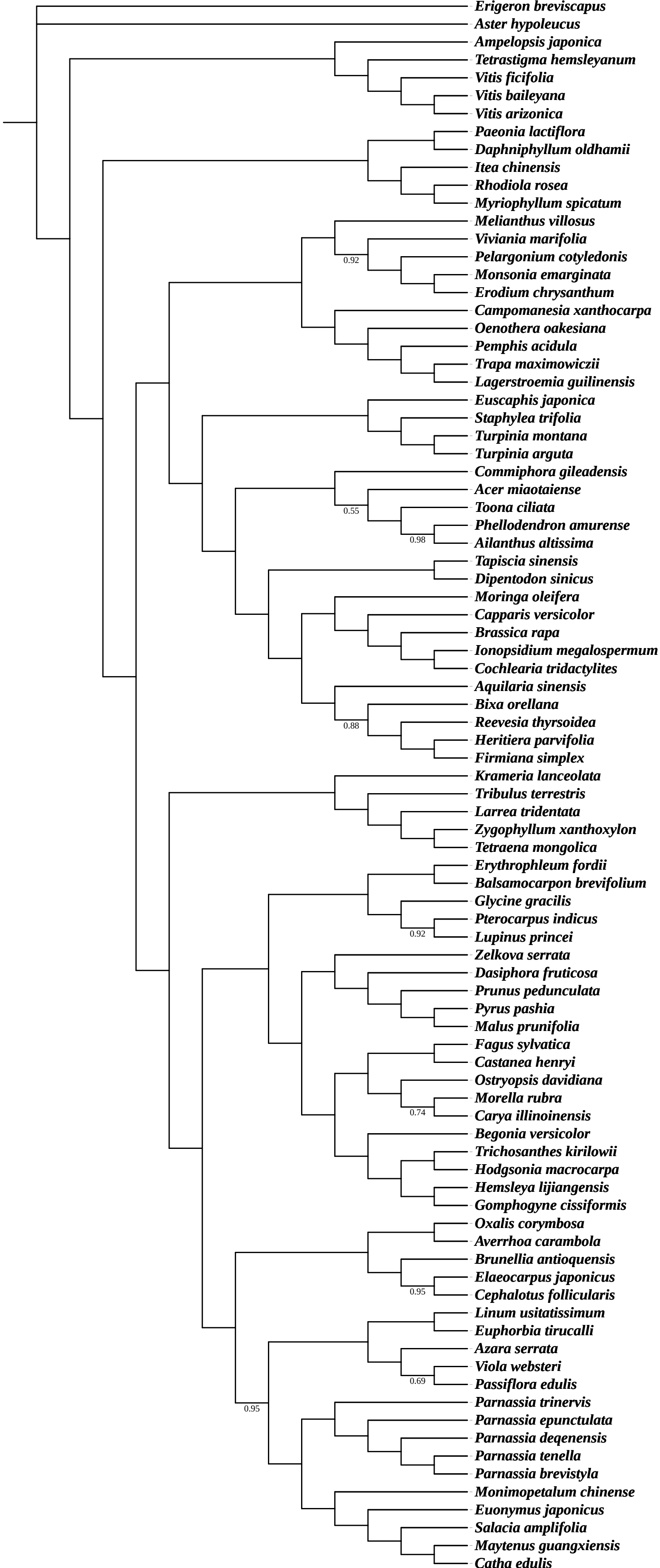

Supplement: Supplementary Figure 3 — Phylogenetic location of Parnassia in Superrosids reconstructed with the dataset based on the first and second sites on the concatenated codons. Numbers associated with branches are Bayesian posterior probabilities. Nodes without numbers indicate posterior probability of 1. [file Image_3.PDF]

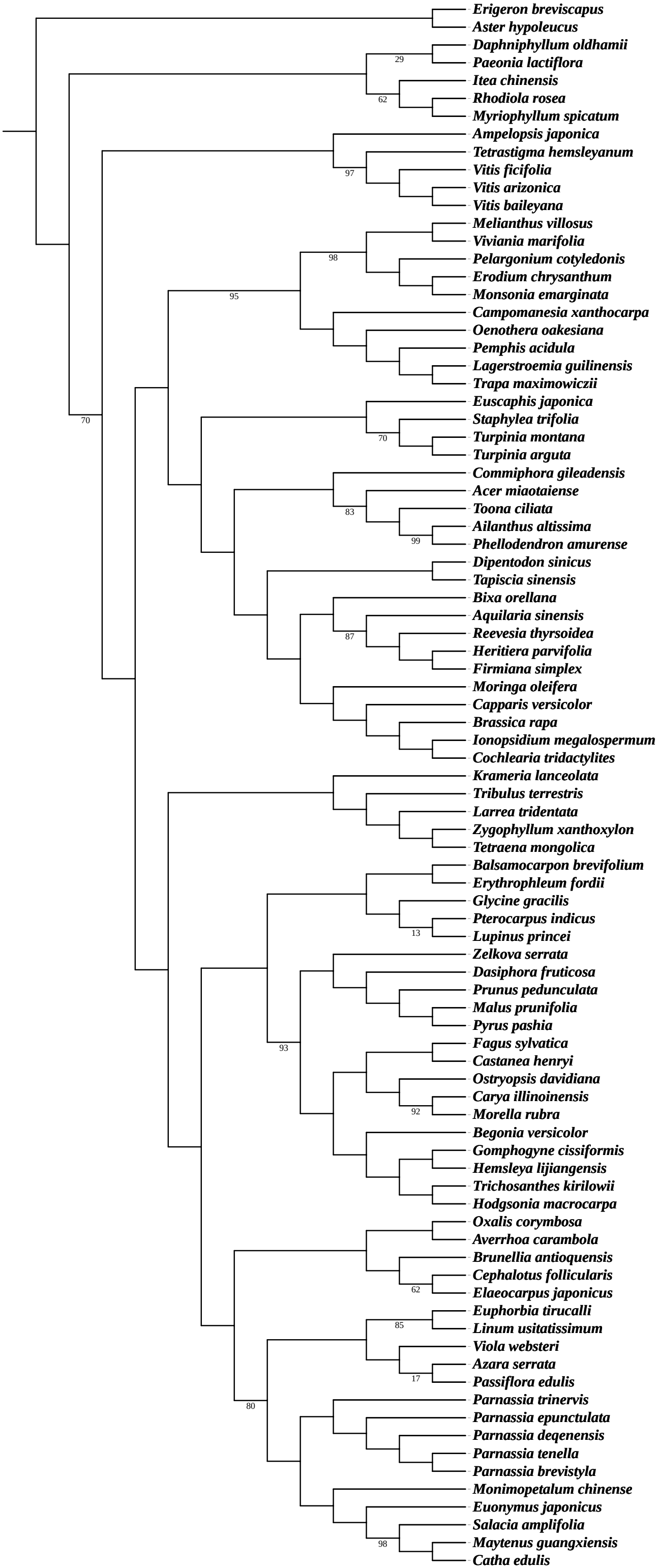

Supplement: Supplementary Figure 4 — Phylogenetic position of Parnassia in Superrosids recovered with the dataset consists of third site on the codons in concatenated. Numbers associated with branches are ML bootstrap values. Nodes without numbers indicate 100% bootstrap support. [file Image_4.PDF]

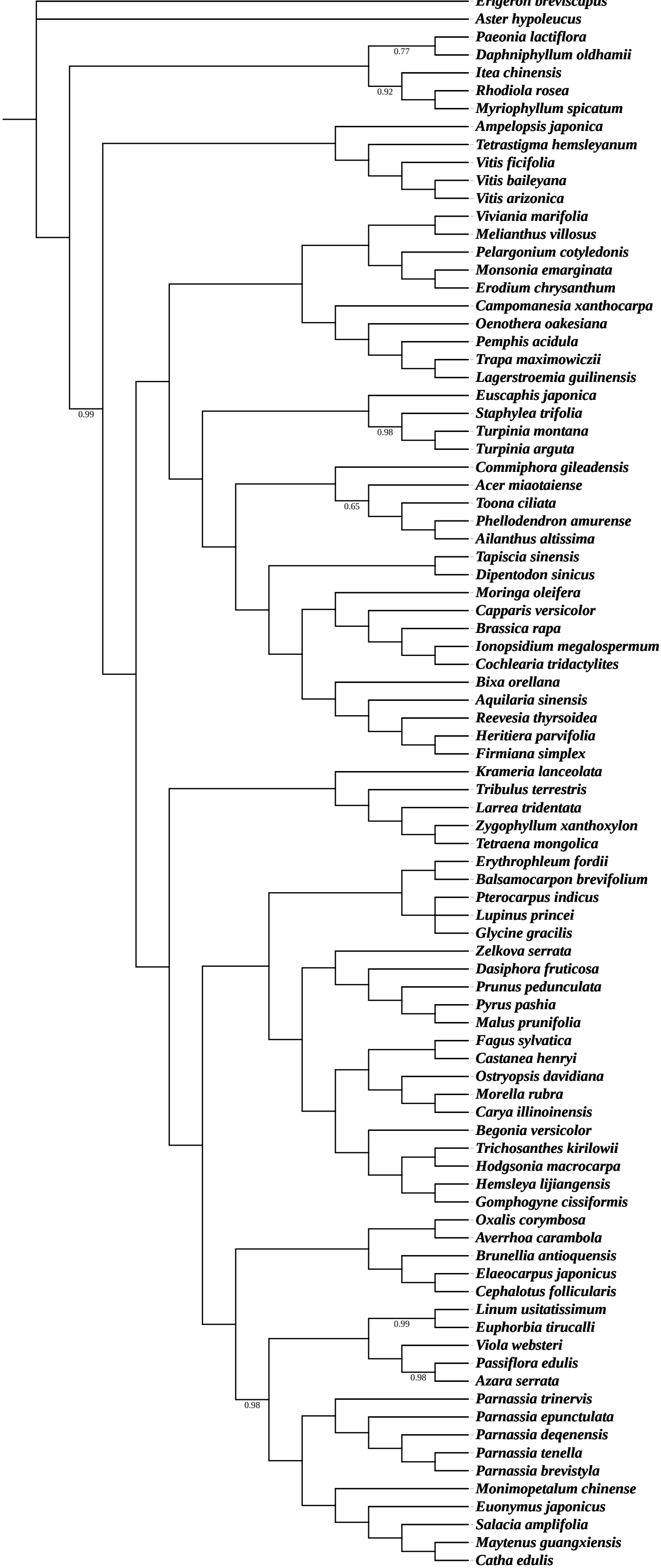

Supplement: Supplementary Figure 5 — Phylogenetic position of Parnassia in Superrosids recovered with the dataset consists of third site on the codons in concatenated. Numbers associated with branches are Bayesian posterior probabilities. Nodes without numbers indicate 1.0 posterior probability. [file Image_5.PDF]

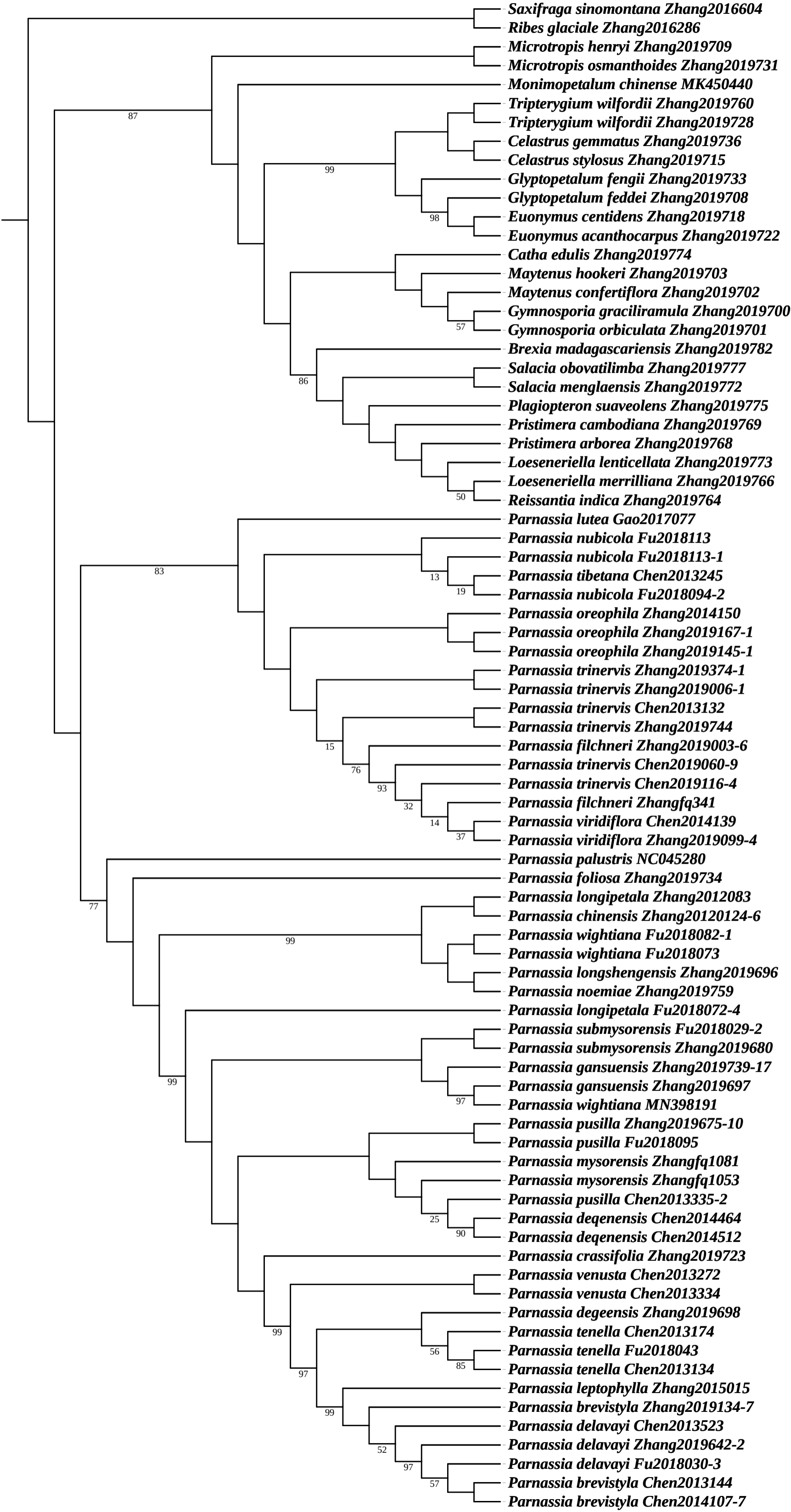

Supplement: Supplementary Figure 6 — Phylogenetic relationship of Parnassia species resolved with the dataset of first and second sites on the concatenated codons. Numbers associated with branches are ML bootstrap values. Nodes without numbers indicate 100% bootstrap support. [file Image_6.PDF]

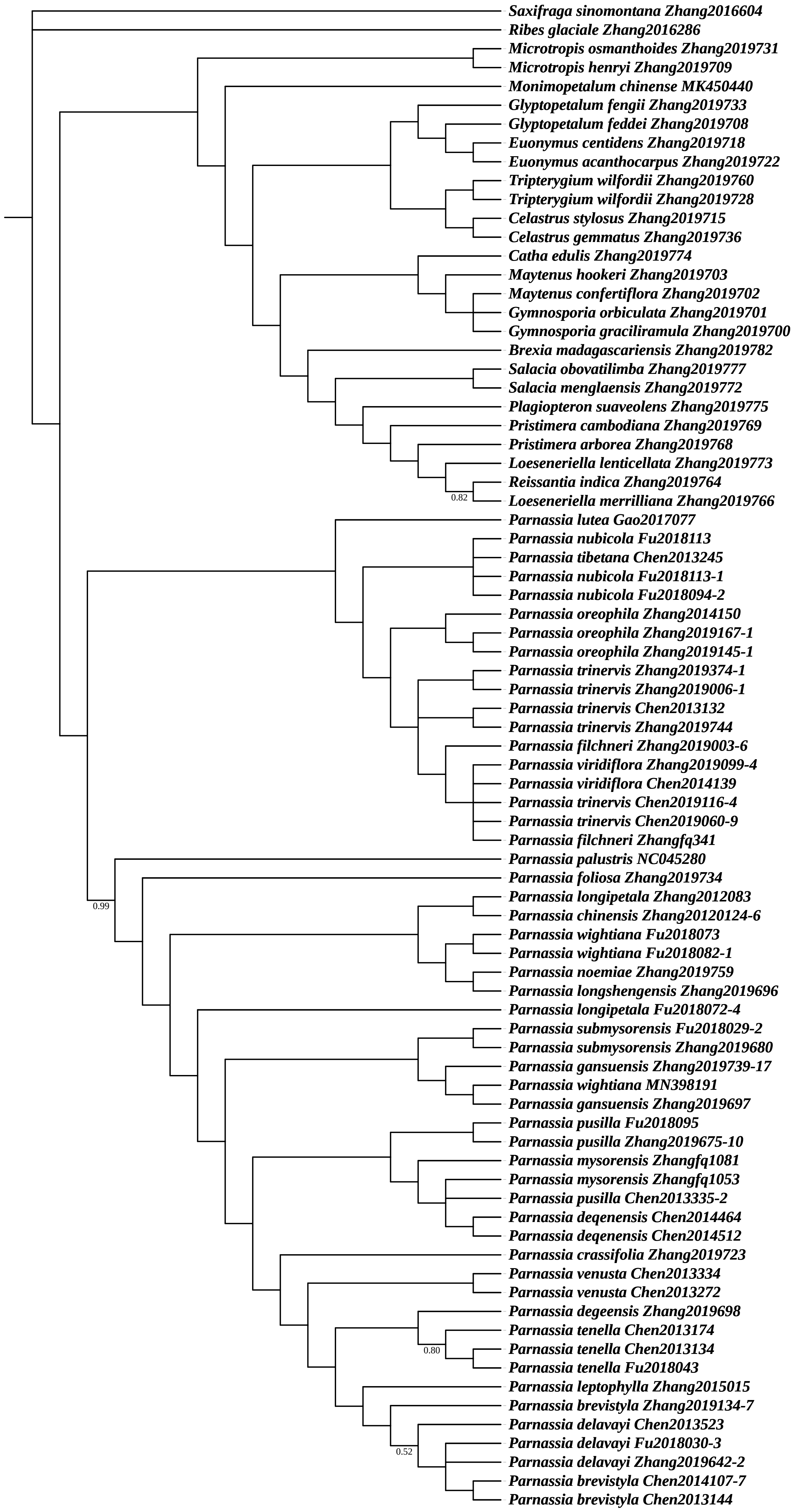

Supplement: Supplementary Figure 7 — Phylogenetic relationship of Parnassia species resolved with the dataset of first and second sites on the concatenated codons. Numbers associated with branches are Bayesian posterior probabilities. Nodes without numbers indicate 1.0 posterior probability. [file Image_7.PDF]

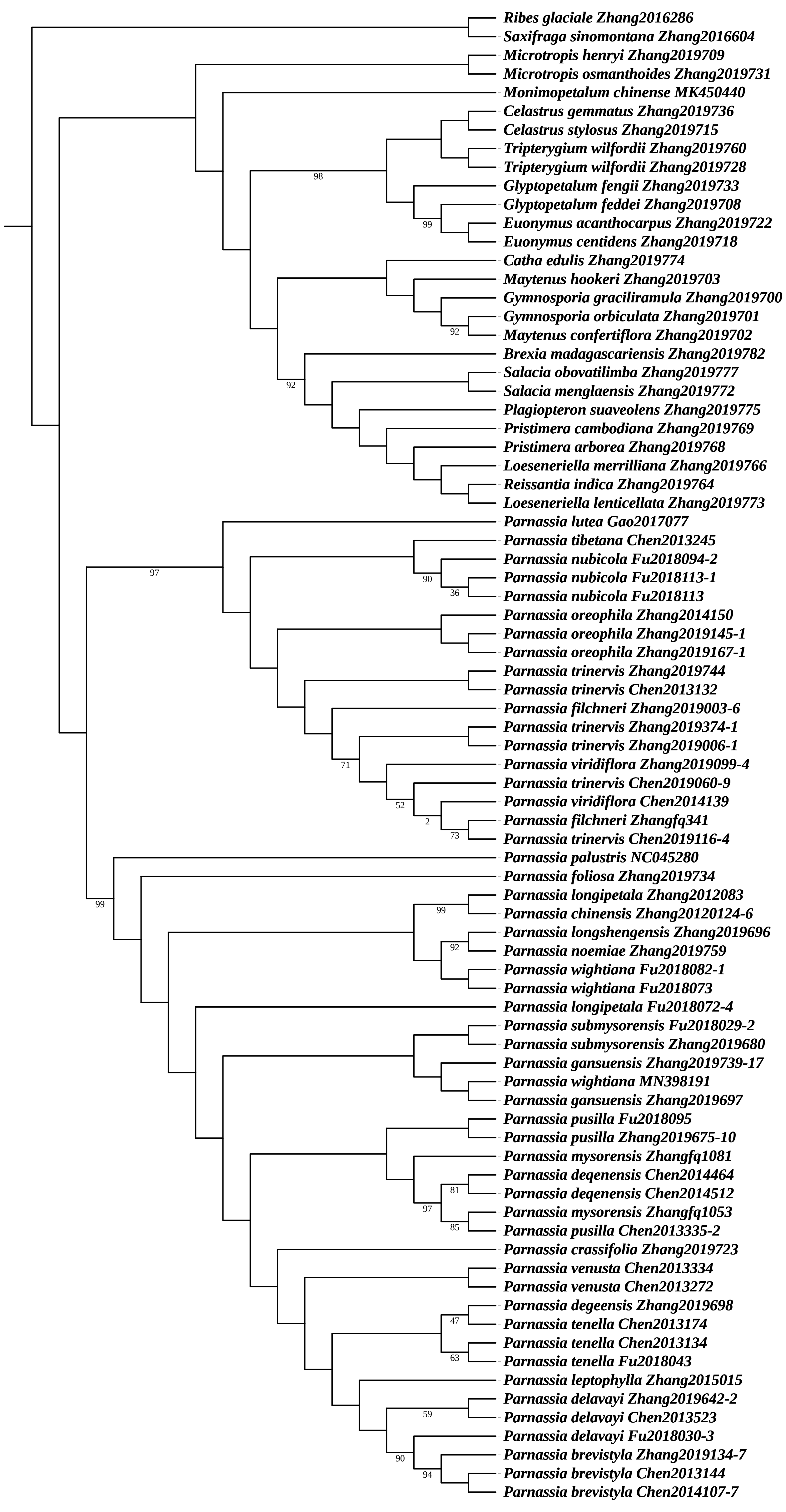

Supplement: Supplementary Figure 8 — Phylogenetic relationship of Parnassia species resolved with the dataset consists of third site on the codons in concatenated. Numbers associated with branches are ML bootstrap values. Nodes without numbers indicate 100% bootstrap support. [file Image_8.PDF]

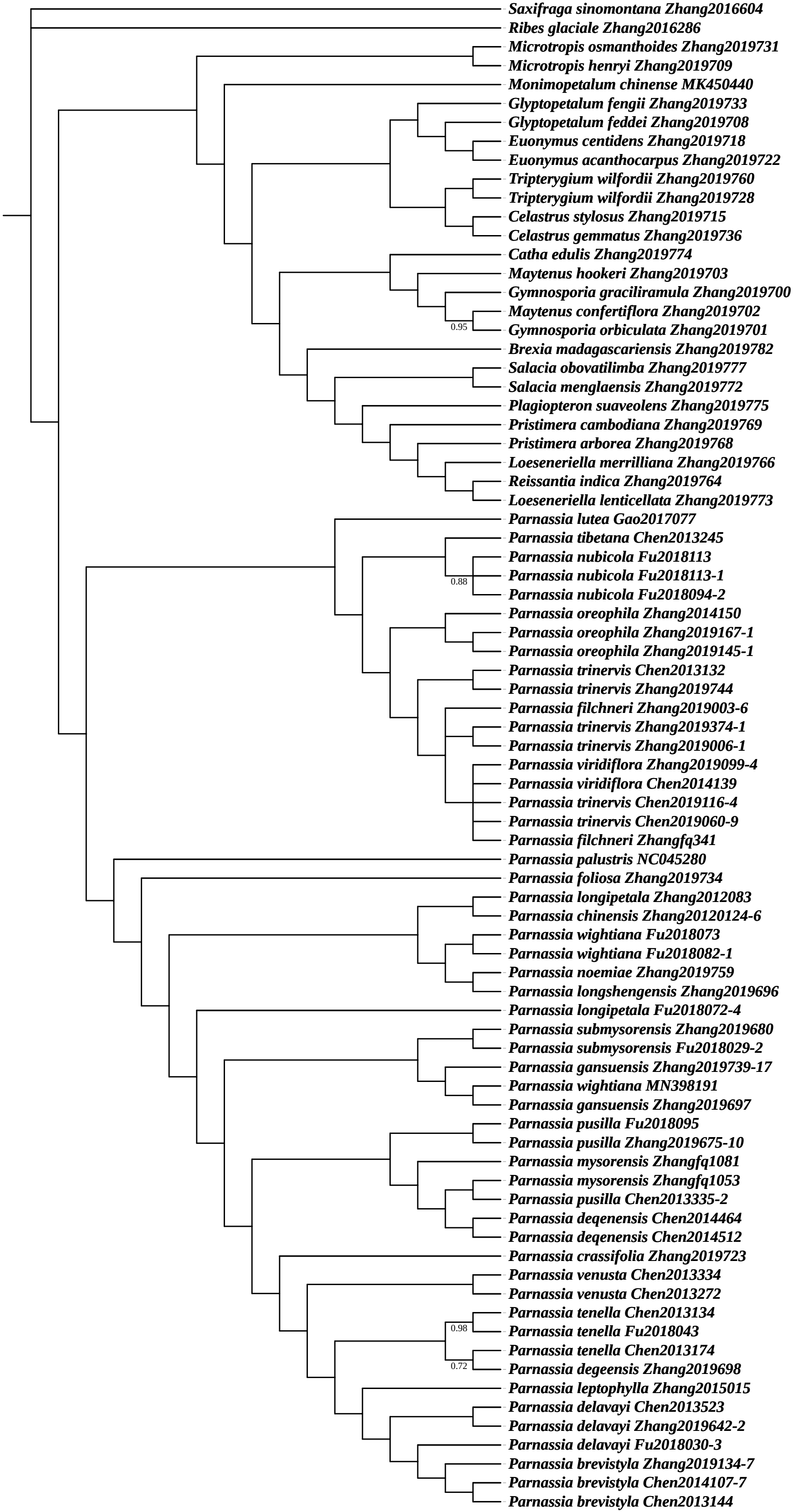

Supplement: Supplementary Figure 9 — Phylogenetic relationship of Parnassia species resolved with the dataset consists of third site on the codons in concatenated. Numbers associated with branches are Bayesian posterior probabilities. Nodes without numbers indicate 1.0 posterior probability. [file Image_9.PDF]

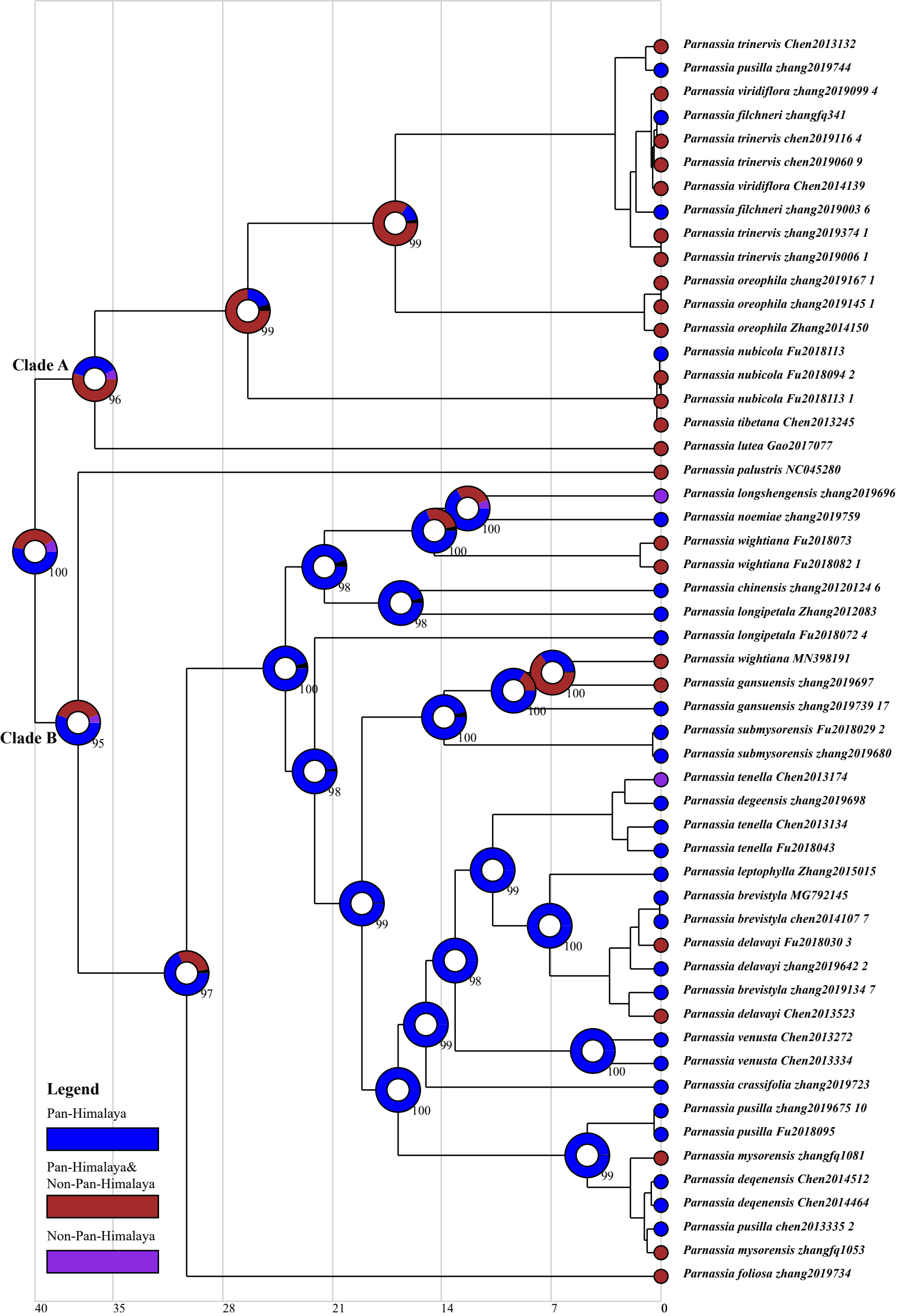

Supplement: Supplementary file 10 [file Image_10.PDF]
